# MALDI-TOF MS for Rapid Analysis of Bacterial Pathogens Causing Urinary Tract Infections in the Riyadh Region

**DOI:** 10.3390/diseases10040078

**Published:** 2022-10-03

**Authors:** Razique Anwer, Hassan Darami, Firas K. Almarri, Mazen A. Albogami, Faisal Alahaydib

**Affiliations:** 1 Department of Pathology, College of Medicine, Imam Mohammad Ibn Saud Islamic University (IMSIU), Riyadh 13317-4233, Saudi Arabia; 2 Department of OB & Gyne, College of Medicine, Imam Mohammad Ibn Saud Islamic University (IMSIU), Riyadh 13317-4233, Saudi Arabia; 3 College of Medicine, Imam Mohammad Ibn Saud Islamic University (IMSIU), Riyadh 13317-4233, Saudi Arabia; 4 Medical Center, Imam Mohammad Ibn Saud Islamic University (IMSIU), Riyadh 13317-4233, Saudi Arabia

**Keywords:** MALDI-TOF MS, *Escherichia coli*, urine samples, UTIs, antimicrobial susceptibility testing

## Abstract

The successful treatment of bacterial disease is relied on selecting a suitable drug based on the type of bacteria and antimicrobial susceptibility testing. The study’s objective was to identify bacterial isolates from urine samples of patients from the community, followed by antimicrobial susceptibility testing of the isolated bacteria. A total of seventy urine samples were received in the clinical microbiology laboratory; out of which 18 culture-positive cultures and by direct identification using MALDI-TOF MS (Matrix Assisted Laser Desorption Ionization Time of Flight Mass Spectrometry) were identified. Of 18 identified bacteria, 17 (94%) were pathogenic. The culture demonstrated that the major species detected in urine samples were *Escherichia coli*, *Klebsiella pneumoniae*, *Enterococcus faecalis*, and *Aeromonas caviae*. *E. coli* (72.2%) was the most common bacterium retrieved from urine samples followed by *K. pneumoniae* (16.6%). Interestingly, all the isolates, except *Enterococcus faecalis*, were resistant to erythromycin. The isolates 8 of 13 (61.5%) were resistant to both of the cotrimoxazole and tetracycline. We performed MLST (Multi-locus Sequence Typing) typing of 13 *E. coli* isolates to study their genetic relatedness and diversity. MLST typing of *E. coli* showed a total of nine different STs (Sequence Types), which showed the diversity among them. ST 129 was the most common ST found in three *E. coli* isolates. In our study, two isolates with ST 1126 and ST 1432 represented the global clonal complex 155. MALDI-TOF MS provided dependable results for identifying the bacteria up to species level from urine samples by indirect culture methods. Such local surveillances are highly recommended for empirical therapy awareness and determining isolates’ level of resistance.

## 1. Introduction

Identification and judgmental diagnosis of the infecting microorganisms are fundamental conditions for efficient treatment and surveillance of nosocomial/community infection. Usually, identification and classification in clinical laboratories are mainly based on phenotypic characteristics, like; Gram stain, growth on different nutrient media, colony morphology, and various biochemical tests, which entail the assignment of a clinical isolate to a genus. In recent years, clinical laboratories have widely adopted molecular biology techniques, like; 16S RNA gene sequencing, Biosensors, and diverse PCR-based techniques to simplify quick and precise identification of a particular pathogen involved in UTIs. Such procedures are laborious and require well-trained technicians to interpret results correctly. However, such methods are not feasible everywhere due to sophisticated procedures and huge costs. Routine biochemical and phenotypic methods like culture and Gram staining are essential for a clinical microbiology laboratory. The culture method for urine samples remains the gold standard for diagnosing a UTI patient. However, following the latter procedure, it takes around 18–36 h to identify bacteria and prescribe a drug to mitigate the UTI.

MALDI-TOF MS (Matrix Assisted Laser Desorption Ionization Time of Flight Mass Spectrometry) technology was introduced recently and has evolved into new clinical diagnostic microbiology applications, showing an innovative, robust, and accurate tool for swift identification of microorganisms [1,2]. It is a rapid, efficient, easy-to-use, and most prominently very cheap method for bacterial identification compared to conventional methods, which saves clinicians the time to start with exact therapy for a critically ill patient. In addition to that, for urine samples, a direct identification (without culture) method has also been utilized by many investigators to get quicker results using MALDI-TOF MS [3,4,5,6,7].

Approximately around 150 million people suffer from UTIs every year, accounting for the most common hospital and community-acquired infections [8]. According to a study, every third woman has been observed with a minimum of one UTI episode requiring antibiotic therapy by 24 years of age [9]. Gram-negative bacteria were the most commonly found pathogens in 75% to 90% of UTI cases. Uropathogenic *Escherichia coli* (UPEC) has been seen in >80% of the community-based UTIs cases, followed by *K. pneumoniae*, *P. mirabilis*, and *P. aeruginosa* [10]. The aforementioned pathogens have also been noticed in the nosocomial infections [11]. Evidently, bacteria causing UTIs are considered the second most frequent form of infection interacted by humans [8]. Notably, women are far more susceptible to UTIs than men; in fact, more than half of all women will acquire a UTI at some point in their life [12]. The mainstays of diagnosing UTIs are mostly by symptoms, laboratory testing, urine dipstick, urine analysis, microscopy, and Gram staining. A significant drawback of identifying uropathogens by these standard methods is that they first must amplify (growth) on agar, which often requires a minimum of 24 h. Luckily, MALDI-TOF-MS is recognized as helpful in several investigations, and it is now being utilized as a standard practice in several laboratories. Within 15 min, MALDI-TOF MS can identify microorganisms in urine, even at bacterial counts as low as 10^3^ CFU [13]. With these unprecedented times with COVID-19, such a piece of advanced equipment can be of use to alleviate the strain on medical laboratories and the government’s healthcare system by providing a prompt identification of infecting organisms. This technology may aid in expediting choosing the appropriate antimicrobial therapy for patients; thereby, reducing the potential adverse effects caused by delayed clearance of microorganisms. So far, not much attention has been paid to the pivotal role of MALDI-TOF-MS in the Middle East, especially in Saudi Arabia. Therefore, this study set out to characterize bacteria that cause urinary tract infections (UTIs) using standard microbiological techniques and MALDI-TOF MS as a unique analysis method. We also performed the multi-locus sequence typing of all the *E. coli* isolates recovered in the present study to investigate their genetic relatedness and diversity.

## 2. Materials and Methods

### 2.1. Urine Collection

From March 2021 to June 2021, we received 70 samples from the Medical Center Microbiology laboratory, Imam University. Approximately 10 mL of midstream urine was aseptically collected in a uricol container from patients and immediately sent for processing to the Biomedical Research laboratory, College of Medicine, Imam University. The collection uricols were labeled with a unique number. The urine samples of patients showing signs and symptoms of UTI were screened with a dipstick test and subjected to further analysis.

CLSI (Clinical and Laboratory Standards Institute) guidelines were followed for performing all laboratory procedures and also our microbiology unit’s standard operation procedure (SOP). Inclusion criteria for this study were applied to adult patients (above 18 years old) presenting with signs and symptoms of urinary tract infection.

### 2.2. Bacterial Culture

Each clinical sample was cultured using the same conventional microbiological procedures. Specimens were collected in the sterile uricols and cultured on the isolation culture media (MacConkey and blood agar (5% defibrinated sheep blood)) followed by incubation at 37 °C. The plates with positive cultures were then inspected under a microscope to assess the colony’s size and morphology. Pure cultures were obtained after streaking isolated colonies for each sample. Gram-stain, colony morphology, and biochemical tests were utilized for initial bacterial identification as per CLSI guidelines and established SOP. Brain and Heart Infusion (SPML) broth vials were prepared with 20% glycerol to store the pure growth of each isolate for further analysis.

### 2.3. Standard Plate Count (SPC)

Blood agar media was used to isolate different types of bacteria from the urine samples. One ml of each of urine sample was mixed with 9 mL 0.1 N NaCl solution, followed by serial dilutions up to 10^−6^ mL. An inoculum of 0.1 mL of each dilution was cultured on blood agar plates by the spread plate method and incubated overnight at 37 °C. The next day, the total number of bacterial colonies on each plate was counted using the standard plate count (SPC) method [14]. A CFU (colony forming unit) of >10^4^/mL in a urine specimen is considered a positive UTI pathogen.

### 2.4. Molecular Analysis by MALDI-TOF MS

Pure bacterial cultures were subjected to molecular identification using MALDI-TOF MS analysis. Standard procedure was followed for processing samples and analyzing the results as used previously [15].

### 2.5. Antimicrobial Susceptibility Tests

The antimicrobial susceptibility testing (AST) of isolates was performed using Kirby–Bauer disk diffusion assay on Mueller–Hinton agar (MHA) plates. The results were interpreted according to CLSI guidelines. Then, 0.5 McFarland bacterium suspension was prepared by picking a pure colony with a sterile straight wire, suspending it in a test tube containing 5–10 mL of 0.1% NaCl solution, and gently mixing until a uniform suspension was formed. Using a sterile cotton swab, the prepared inoculum was streaked as lawn culture on dried Mueller–Hinton agar plate. The inoculated plates were left to air dry at an inverted position for 10 min, approximately covered with the lid. A panel of antibiotic disks was placed on inoculated plates using sterile forceps or needles. Seven antimicrobial agents from different classes were used: nitrofurantoin, tetracycline, cotrimoxazole, erythromycin, amikacin, gentamicin, and tobramycin. The discs were set at least 24 mm apart and 15 mm from the edge to prevent overlapping the zone of inhibition. The plates were incubated overnight (~18 h) at 37 °C. The next day, the inhibition zone diameter around each antibiotic disk was measured. The susceptibility report was prepared for each bacterial isolate as S (susceptible), R (resistant), or I (intermediate) for every antibiotic by comparing them with the standard zones given in the CLSI guidelines.

### 2.6. DNA Isolation

Overnight grown cultures of *E. coli* isolates were subjected to DNA isolation using the heat shock method. A loop full of culture was mixed with 100 µL of nuclease-free water in a 200 µL tube. The prepared mixture was placed at −20 °C for 20 min. Following this, the mixture tube was immediately placed at 95 °C for 15 min using Polymerase Chain Reaction (PCR) machine. The mixture was centrifuged at 8000× *g* for 1 min and the supernatant was pipetted out, which was used as DNA samples for PCR amplification further. Isolated DNA integrity and concentration was checked using a Nanodrop spectrophotometer.

### 2.7. PCR & MLST Analysis

PCR amplification was done for seven housekeeping genes (*adk*, *fumC*, *gyrB*, *icd*, *mdh*, *purA*, and *recA*) of 13 isolates of *E. coli* (pubmlst.org/bigsdb?db=pubmlst_ecoli_achtman_seqdef). Primers for seven housekeeping genes were synthesized and procured from Sigma Aldrich. Agarose gel electrophoresis was used to run the amplified product of each gene. PCR product size was verified for each gene using appropriate band length in the gel documentation system. Gel purification was performed after cutting the gel portion having the amplicon. DNA reading was taken for the purified amplicons and sent for DNA sequencing. After sequencing, dendrograms were checked to confirm the quality of the sequenced genes using DNASTAR software. Allelic profiles of each *E. coli* isolate were generated using the PubMLST database. STs were assigned to *E. coli* using their allelic profiles at PubMLST database.

### 2.8. Phylogenetic Tree Analysis

Concatenated sequence files were prepared using FASTA format of each gene for each isolate. Seven gene sequences (concatenated) were placed in a single FASTA file for all the isolates. ClustalX2 was used to perform multiple sequence alignment, which was saved in the .aln file. This alignment file was opened in MEGA 6 version software and converted to .meg file. Using .meg file, the ‘Maximum Likelihood’ and Neighbor-joining phylogenetic trees were constructed by MEGA 6.

### 2.9. Data Analysis

Breakpoints given in the CLSI guidelines (CLSI M23Ed5) were referred for interpretation of the AST results [16]. ATCC strains of *P. aeruginosa*, *E. coli*, and *S. aureus* were used as reference strains for the quality check of performed culture and susceptibility testing procedures.

## 3. Results

**Figure 1:**
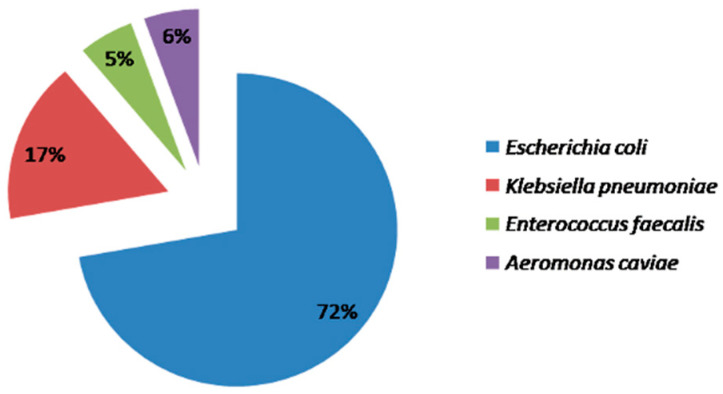
Percentage of isolated UTI pathogens.

A total of 70 samples were collected and subjected to growth on culture media and susceptibility tests, giving a 25.7% response rate. Out of 70 samples, 18 showed significant growth of bacteria with CFU < 5000 bacteria/mL. If a CFU of ≥10^5^/mL is traced in a midstream urine sample, a confirmed case of UTI with bacteriuria is considered. Most of the study cases live in Riyadh’s urban area. In the present study, four different bacteria were identified in a total of 18 samples, and all were GNB (Gram-negative Bacteria) except one (*E. faecalis*). *E. coli*, *K. pneumonia*, *E. faecalis*, and *A. caviae* were the four major genus detected in all the urine samples (Table 1). *E. coli* (72.2%, 13/18) were the most common bacteria retrieved from urine samples, followed by *K. pneumoniae* (16.6%, 3/18) (Figure 1).

One isolate of *E. faecalis* and *A. caviae* was traced (Table 1). Interestingly, all the isolates, except *Enterococcus faecalis*, were resistant to erythromycin. Out of 13 isolates, eight were resistant to cotrimoxazole and tetracycline (Table 1). All the isolates were susceptible to amikacin, gentamycin, tobramycin, and nitrofurantoin (Table 1).

In this study, all 18 culture-positive samples were subjected to MALDI TOF, which correctly identified the isolates up to species level. MALDI TOF provided a score list of all the bacterial species with moderate to highest scores of 1.8 to 2.2. All the bacteria were correctly identified compared to the biochemical and morphological test results. There was not a single false result or misidentification by MALDI TOF. Many previous studies have identified bacterial pathogens from direct urine samples. However, they failed to recognize all the samples grown in culture methods and later identified MALDI-TOF MS. However, we got the fine results with indirect culture methods in the current study. A total of nine Sequence Types (STs) were assigned to 13 *E. coli* isolates by MLST typing, which showed the diversity among the latter (Table 2).

**Figure 2:**
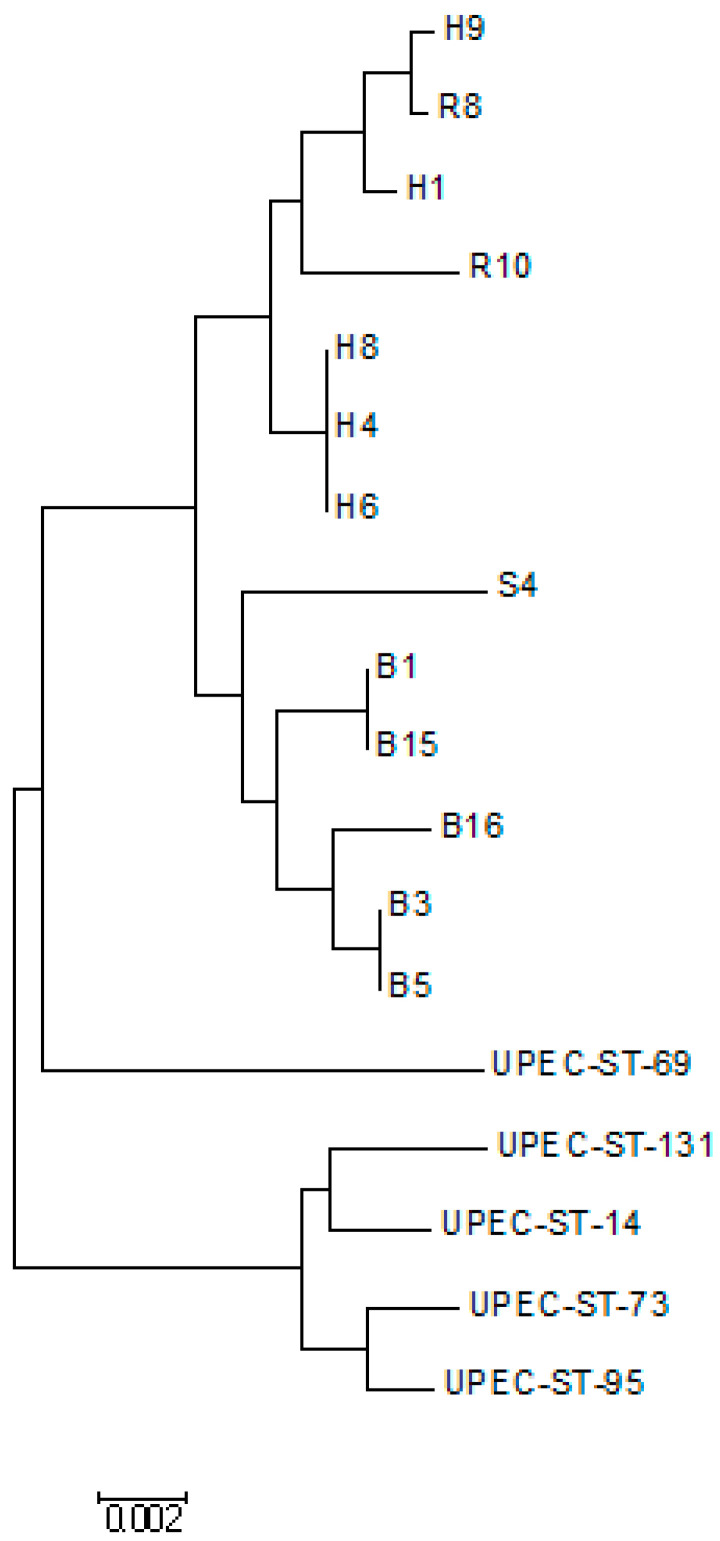
Molecular phylogenetic analysis by Maximum Likelihood method.

**Figure 3:**
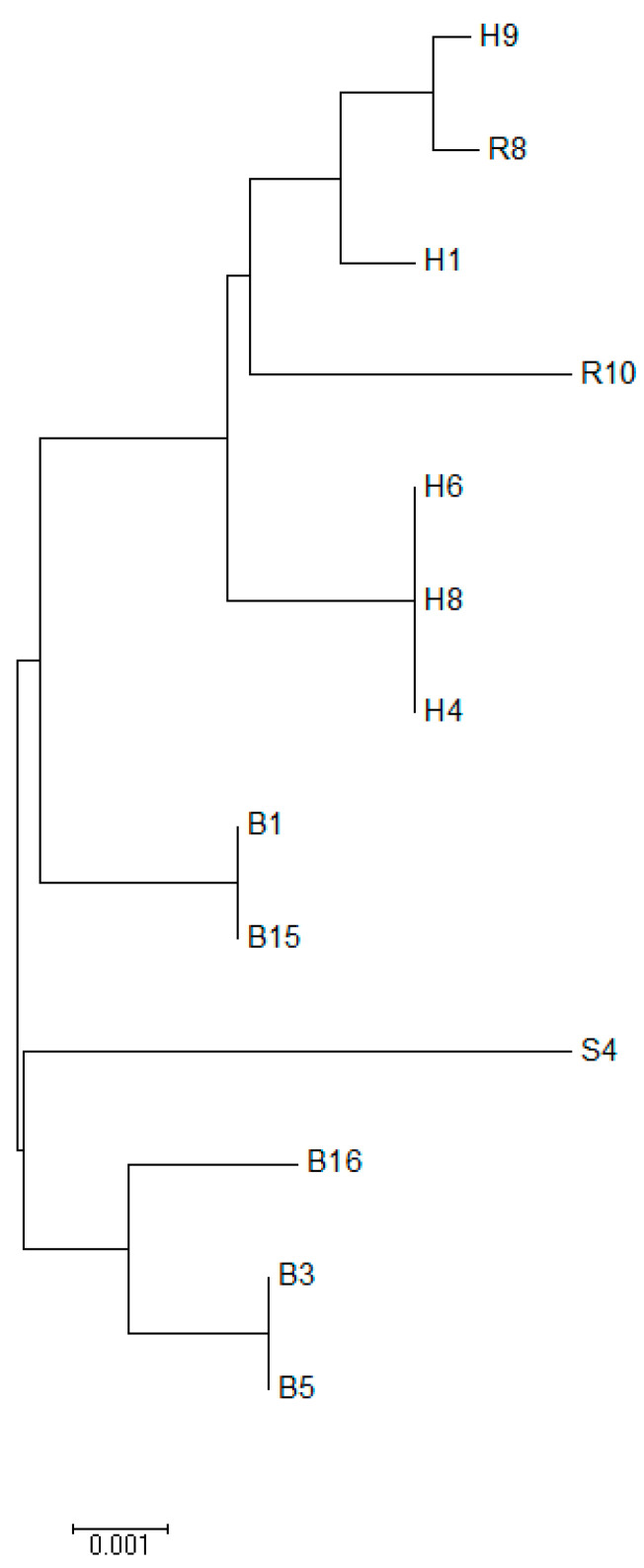
Evolutionary relationships of taxa.

ST 129 was the most common ST found in three *E. coli* isolates. ST 2033, ST 1015 were found in two *E. coli* isolates each. Two isolates with ST 1126 and ST 1432 represented the global clonal complex (CC) 155. Maximum-Likelihood tree and Neighbor-Joining phylogenetic trees showed a high genetic diversity among all the *E. coli* isolates. Maximum Likelihood tree showed two different clads in the *E. coli* population (Figure 2 and Figure 3).

The evolutionary history was inferred using the Maximum Likelihood method based on the Tamura-Nei model [17]. The tree with the highest log likelihood (−5402.1141) is shown. Initial tree(s) for the heuristic search were obtained automatically by applying Neighbor-Join and BioNJ algorithms to a matrix of pairwise distances estimated using the Maximum Composite Likelihood (MCL) approach, and then selecting the topology with superior log likelihood value. The tree is drawn to scale, with branch lengths measured in the number of substitutions per site. The analysis involved 18 nucleotide sequences. Codon positions included were 1st + 2nd + 3rd + Noncoding. All positions containing gaps and missing data were eliminated. There was a total of 3413 positions in the final dataset. Evolutionary analyses were conducted in MEGA6 [18]). UPEC isolate with ST-14, ST-69, ST-73, ST-95, and ST-131 were taken as standard strains to compare the genetic relatedness.

The evolutionary history was inferred using the Neighbor-Joining method [19]. The optimal tree with the sum of branch length = 0.02339287 is shown. The tree is drawn to scale, with branch lengths in the same units as those of the evolutionary distances used to infer the phylogenetic tree. The evolutionary distances were computed using the Maximum Composite Likelihood method [20] and are in the units of the number of base substitutions per site. The analysis involved 13 nucleotide sequences. Codon positions included were 1st + 2nd + 3rd + Noncoding. All positions containing gaps and missing data were eliminated. There was a total of 3423 positions in the final dataset. Evolutionary analyses were conducted in MEGA6 [18]).

Herein, using MALDI-TOF MS technology, we identified *E. coli* (13/18) being the dominating microorganisms, followed by *K. pneumoniae* (3/18), *E. faecalis* (1/18), and *Aeromonas caviae* (1/18). Out of four types traced, three were pathogenic bacteria with a total count of 17.

## 4. Discussion

The most common reason for prescribing antibiotics is urinary tract infections, and early detection can allow for rapid antibiotic treatment and prevent complications. The time between receiving a sample and identifying the pathogen is roughly 24 to 48 h, which might be significantly reduced if a reliable direct technique was used [21]. MALDI-TOF MS works on the principle of identification of the protein profile of a microorganism, which is particularly assigned to a specific microbial species. MALDI-TOF MS gives the most accurate, rapid, and affordable bacterial/microbial identification results in clinical laboratory settings [22]. Direct microbial identification using MALDI-TOF MS has also been used for many clinical samples such as blood, urine, CSF, and wound swabs. Compared to molecular techniques, MALDI-TOF MS is an easier, time-saving, and cost-effective technique used in microbiology labs. Herein, we detected the pathogenic bacteria from urine samples from UTI patients using an indirect culture-based method using MALDI-TOF MS.

In the present study, we aimed to identify bacterial pathogens in the midstream urine samples using MALDI-TOF MS-based followed by antimicrobial susceptibility testing. We reported 17/18 as potential pathogenic bacteria with different susceptibilities to various antibiotics. As reported in the previous investigation, we got accurate results using the MALDI-TOF MS platform for indirect culture-based identification [23]. Previously, many studies have reported rapid identification using the MALDI-TOF MS platform and compared it with conventional methods. Pioneered by Ferreira et al. [3], they have established a direct identification method by MAL-DI-TOF MS; they identified *E. coli* from the urine samples in 94.2% of cases (*n* = 163). Using MALDI-TOF MS and flow cytometry, Wei et al. developed a new method of directly identifying microbial pathogens from urine samples. This study used MALDI-TOF MS to directly identify 18.7% (*n* = 307) of urine samples driven by bacterial pellets. Direct identification revealed 43.23% *E. coli* (*n* = 99), 15.28% *K. pneumoniae* (*n* = 35), and 13.97% *Enterococcus* spp. (*n* = 32) as the most common bacteria in the study. Another study demonstrated 88.59% GNB (*n* = 163), which had a score of more than 2, 9.24% (*n* = 17) had a score between 1.7 and 2, and 2.17% (*n* = 4) had a score less than 1.7 [7], which is quite similar to our study.

Previously, MALDI-TOF MS was only used to detect the etiological agent, and conventional methods were used to study antibiotic susceptibility tests and their resistivity. Many methods have been previously proposed based on MALDI-TOF MS for detecting antimicrobial resistance and antibiograms for several bacterial species [24]. Activities of β-lactamase were assessed using MALDI-TOF MS, which showed the decreasing pattern of mass spectra peaks representing hydrolyzing activity of β-lactamase in response to β-lactam antibiotics [25]. Using MALDI-TOF MS, Johansson et al. developed methods to detect carbapenemase production in *Bacteroides fragilis* strains encoding the cfiA gene [26]. Class C beta-lactamase of *Acinetobacter baumannii*, which belongs to the extended beta-lactamase of spectrum ADC family, has recently been detected by MALDI-TOF MS, which can be used as carbapenem resistance biomarker [27]. As a result of these studies, the number of antibiotics that can be used empirically has been significantly reduced. It is predicted that mechanisms responsible for antimicrobial resistance, such as porins, efflux transporters, and similar others can be identified using MALDI-TOF in future investigations. MLST analysis is crucial in determining genetic relatedness or divergence in a population under investigation. The present study showed a significant diversity in the *E. coli* population. In 2020, another study from the same region pointed out CC 131 of *E. coli* in the UTI samples by multiplex PCR method [28]. However, our study highlighted CC 155, which suggested the circulation of more *E. coli* clones in the region. However, the number of isolates was not very much, but this study pointed out the need for alarge-scale study. Another study from Saudi surfaced 32 STs using the MLST typing method in 2018 [29]. This study also highlighted CC 131, followed by CC 38. The diagnosis of UTI within a few minutes using MALDI-TOF allowed more precise confidence for initiating empirical therapy. In brief, UTI pathogens can be easily treated provided a combined system of MALDI-TOF MS and antimicrobial susceptibility detector.

Some limitations of our study should be considered. The study was conducted in the capital of Saudi Arabia, Riyadh, where most of the population is educated and aware of good hygiene practices and antibiotics usage; hence, future studies in other regions of Saudi Arabia are required. Additionally, the number of isolates being small, it raises a question of the validity of the results. Therefore, further studies with a larger sample size are needed.

## 5. Conclusions

The present study showed a high prevalence of pathogenic bacteria (25.7%) in urine samples, causing UTIs where most commonly used antibiotics were ineffective. *E. coli* and *K. pneumoniae* were the most commonly found bacterial isolates. The least resistant antibiotics for Gram-negative isolates were gentamycin, amikacin, tobramycin, and nitrofurantoin. On the other hand, a high resistance level was seen for erythromycin, cotrimoxazole, and tetracycline. MLST analysis showed high genetic diversity among the *E. coli* isolates. Such surveillance programs are recommended further for routine practice of empirical therapy for UTI. Therefore, monitoring of resistance mechanisms and a proper stewardship program are necessary.

## Figures and Tables

**Table 1:** MALDI results and Antimicrobial Susceptibility Testing (AST) profile of urine isolates.

S. No.	Isolate ID	MALDI Results	Source	MALDI Score	Nitrofurantoine	Cotrimoxazole	Tetracycline	Erythromycin	Amikacin	Gentamicin	Tobramycin
1	B-1	*E. coli*	Urine	2.26	S (26)	S (31)	S (24)	R	S (24)	S (27)	S (32)
2	B-15	*E. coli*	Urine	1.9	S (21)	R	R	R	S (21)	I (14)	S (15)
3	B-16	*E. coli*	Urine	1.56	S (23)	S (30)	R	R	S (25)	S (26)	S (19)
4	B-3	*E. coli*	Urine	2	S (31)	R	R	R	S (23)	S (25)	S (25)
5	B-5	*E. coli*	Urine	2.1	S (31)	R	R	R	S (24)	I (14)	S (17)
6	H-1	*E. coli*	Urine	1.89	R	R	S (19)	R	S (21)	S (26)	S (27)
7	H-10	*Enterococcus faecalis*	Urine	1.9	S (31)	S (31)	R	S (30)	R	S (26)	S (24)
8	H-4	*E. coli*	Urine	1.87	S (26)	R	R	R	S (24)	S (25)	S (25)
9	H-5	*Klebsiella pneumoniae*	Urine	1.77	S (26)	S (34)	S (25)	R	S (31)	S (29)	R
10	H-6	*E. coli*	Urine	1.41	S (21)	R	R	R	S (21)	S (21)	S (21)
11	H-7	*Klebsiella pneumoniae*	Urine	1.5	S (24)	S (31)	S (17)	R	S (27)	S (27)	S (27)
12	H-8	*E. coli*	Urine	1.4	S (27)	S (35)	R	R	S (29)	S (27)	S (27)
13	H-9	*E. coli*	Urine	2	S (27)	R	R	R	S (26)	S (26)	S (26)
14	R-5	*Aeromonas caviae*	Urine	2.15	S (25)	S (25)	S (17)	R	S (26)	S (27)	S (26)
15	R-8	*E. coli*	Urine	1.89	S (25)	S (27)	S (17)	R	S (24)	S (25)	S (26)
16	R-10	*E. coli*	Urine	1.63	S (27)	R	S (20)	R	S (25)	S (25)	S (26)
17	S-3	*Klebsiella pneumoniae*	Urine	1.4	S (18)	S (28)	S (20)	R	S (24)	S (26)	S (26)
18	S-4	*E. coli*	Urine	1.69	S (24)	S (28)	S (17)	R	S (25)	S (26)	S (25)

**Table 2:** MLST profile of 13 *E. coli* isolates.

S. No.	*E. coli* ID	MLST Allele Profiles of Each Gene							ST
		*adk*	*fumC*	*gyrB*	*icd*	*mdh*	*purA*	*recA*	
1	B1	6	6	15	10	9	7	14	2033
2	B15	6	6	15	10	9	7	14	2033
3	B16	6	4	4	16	24	5	14	1126
4	B3	6	4	14	16	24	13	14	1015
5	B5	6	4	14	16	24	13	14	1015
6	H1	6	4	5	18	11	8	14	533
7	H4	6	23	3	26	9	7	7	129
8	H6	6	23	3	26	9	7	7	129
9	H8	6	23	3	26	9	7	7	129
10	H9	6	65	4	18	24	8	14	1432
11	R8	6	4	4	18	176	8	14	1493
12	R10	6	6	15	56	8	26	6	1987
13	S4	6	11	4	16	11	7	2	1773

## Data Availability

All datasets generated or analyzed during this study are included in the manuscript.
